# Health Tract, No. 132

**Published:** 1863-04

**Authors:** 


### HEALTH TRACT, No. 132.

### SCHOOL-CHILDREN.

This beautifully bright morning of March the fifth, with the thermometer at within twelve of zero of Fahrenheit, at eight o'clock, found us taking the usual walk of a mile and a half along Fifth Avenue, from dwelling to office, with our four responsibilities, who go to school near by. Alice, our eight-year old, who was full of talk, said: "Father, I wish I was my teacher's pet, but I am not; her pets can do as they please, but she is so strict with the rest of us." "Who are her pets, my daughter?" "The ones that know their lessons best." "Are they larger or smaller than you?" "Oh! they are the tiniest girls in the school. My teacher says the smallest girls in the school are the smartest."

On another occasion, when told of a girl who was never absent, never missed a word in any of her lessons, I inquired if she was good-looking. The reply was: "She is so pale and thin; and there are sores on her hands and face." Similar answers have been made in various other cases. The actual fact is, that the good scholars study themselves to death, and are petted and favored in a great variety of ways; while those of less mental capacity are treated with an impatience and a sternness which soon gives them a dislike for school, for their teachers and for learning in general, and Saturdays and Sundays are the only sunshiny days of the week to them. I frequently say to my children: I don't want you to strive for "head." I don't want you to be promoted, for the oftener you are, the harder you will have to study. You have plenty of time, and I would rather see you eat heartily, and sleep soundly, and know but little, than that you should know a great deal, and grow pale, and thin, and weakly, and die before you are grown up.

Among the most important observances for school-children, and which every wise and affectionate parent will never lose sight of, are,

1st. See that they have all the sleep they can take. Every child under ten should be in bed by eight o'clock, summer and winter, so that they may have nearly eleven hours' sleep. Those older, should be in bed at nine and be required to rise at six; thus they will have more time for study in the morning, when the brain is rested and acts efficiently, and will also be prevented from injuring their eyes, as very many school-children do, by using artificial light.

2d. See to it that every child goes to bed with warm, dry feet, and that they sleep warm all night.

3d. If you are a human, and not a brute, never allow your child to go to bed with wounded or ruffled feelings from any angry words, or harsh or hasty conduct on your part. Always send them off to school in a happy and affectionate state of mind; and when they return, let them be invariably received with a kindly greeting, and a loving, thankful heart that they are once more returned to you in health and safety. These things are the more necessary as their ambitions, their disappointments, their discouragements, and their troubles, in reference to their school and their lessons, are as important to them as yours to you in the mightier matters of life, and if they find not a balm for all these in the affection, and smiles, and sympathy, of their mothers especially, it is to them a misfortune, and to such mothers a disgrace.

4th. By all possible means arrange that your children shall reach school with dry feet and dry clothing; the neglect of this has sent many a sweet child to its early grave, the victim of a mother's carelessness or a teacher's stupidity.

5th. School-children should eat with great regularity; thrice a day is all-sufficient for those above ten. Frequent eating, and tempting their appetites with sweetmeats and delicacies, has been the ground-work of early and life-long dyspeptics to multitudes.

6th. Teach children perseveringly the importance of attending promptly to the calls of nature; and by any and every means bring it about that this shall be done before leaving for school in the morning. To this end arrange that they shall be through with their breakfasts an hour before it is necessary to start for school, even if they have to eat by candle-light. Cases of fatal inflammation of the bladder have often occurred in consequence of the ignorance or brutality of teachers in this connection.

7th. Embrace every opportunity of impressing the child's mind with the fact that teachers are laboring for their good, and therefore ought to be loved, respected, and obeyed, as their best friends.

